# Clinical implication of the BRAF^V600E^ mutation in papillary thyroid carcinoma

**DOI:** 10.1186/1477-7819-11-99

**Published:** 2013-05-20

**Authors:** Yong-Seok Kim, Jeong-Soo Kim, Ja-Seong Bae, Woo-Chan Park

**Affiliations:** 1 Department of Surgery, College of Medicine, The Catholic University of Korea, Seoul, Korea

**Keywords:** BRAF mutation, Papillary carcinoma, Thyroid

## Abstract

**Background:**

The BRAF^V600E^ mutation is the most common genetic alteration in papillary thyroid carcinoma (PTC). In recent studies, the BRAF^V600E^ mutation has been associated with poor clinicopathological characteristics, such as lymph node metastasis, extrathyroidal extension, and advanced stage. However, other studies have failed to establish an association between the BRAF^V600E^ mutation and clinicopathological features. Therefore, we investigated the relationship between the BRAF^V600E^ mutation and its clinicopathological factors at a single institution.

**Methods:**

A total of 327 consecutive patients with PTC were enrolled in this study and underwent thyroid surgery at Yeouido St. Mary’s Hospital between February 2010 and December 2011.

BRAF^V600E^ mutation analysis was performed using polymerase chain reaction (PCR)-based amplification of DNA extracted from paraffin-embedded tumour specimens.

**Results:**

The BRAF^V600E^ mutation was detected in the tumours of 241 (73.7%) patients. Lymph node metastasis, TNM stage, and multifocality were not significantly associated with the BRAF^V600E^ mutation. However, larger tumour size, extrathyroidal extension, histologic type (classic type), and concurrent Hashimoto’s thyroiditis were associated with the BRAF^V600E^ mutation in the univariate analysis, although no clinicopathological features were associated with the BRAF^V600E^ mutation in the multivariate analysis.

**Conclusion:**

There was no idependent prognostic factor associated with BRAF^V600E^ mutation status in this study. The BRAF^V600E^ mutation is unlikely to serve as a prognostic factor for PTC.

## Background

Papillary thyroid carcinoma (PTC) is the most common endocrine malignancy, accounting for 85% to 90% of all thyroid malignancies [1,2]. PTC is generally treated successfully with surgery, radioiodine ablation, and levothyroxine suppression therapy. This malignancy typically has a favourable prognosis, with an average 10-year survival rate of over 90%, although up to 35% of patients suffer from disease recurrence during long-term follow up [3].

Several genetic events have been associated with PTC, and the BRAF mutation is the most common genetic alteration detected in patients with PTC [4]. The RAF protein has three isoforms, with BRAF being the most common isoform found in thyroid follicular cells and the strongest activator of mitogen-activated protein kinase signalling [5]. The most common BRAF mutation in thyroid cancer, occurring in more than 95% of cases, is the T1799A transversion mutation in exon 15. The thymidine to adenine transversion at position 1,799 results in a valine to glutamic acid substitution at residue 600 (V600E) [4,6] and occurs at a prevalence ranging from 29% to 83% [7,8].

The BRAF^V600E^ mutation in PTC has been investigated at many institutions, but the relationship between the BRAF^V600E^ mutation and the clinicopathological features of PTC remain controversial. Several studies have reported that the BRAF^V600E^ mutation is associated with a poor prognosis when combined with the aggressive clinicopathological features of PTC [8,9], whereas other studies have failed to demonstrate a statistically significant association between the BRAF^V600E^ mutation and aggressive clinicopathological features [7,10]. Therefore, the purpose of this study was to evaluate the prevalence of the BRAF^V600E^ mutation in PTC and to analyse the association between the BRAF^V600E^ mutation and the clinicopathological features of PTC at a single institution.

## Methods

### Patients

A total of 327 consecutive patients with PTC were enrolled in this study and underwent thyroid surgery at Yeouido St Mary’s Hospital of the Catholic University in Seoul, Korea, between February 2010 and December 2011. We routinely performed central lymph node dissections in all patients and selectively performed lateral neck lymph node dissections if the preoperative imaging studies (ultrasound and computed tomography) raised suspicions of malignancy and if the fine needle aspiration cytology (FNAC) were found to contain atypical cells or metastatic papillary carcinomas. The patients diagnosed with a recurrence of PTC and those who refused to be enrolled were excluded. Written informed consent was obtained from all patients in advance. This study was reviewed and approved by the institutional review board at Yeouido St Mary’s Hospital (SC13TISI0007).

### DNA extraction

DNA was extracted from five paraffin sections with a thickness of 10 μm that contained a representative portion of the tumour tissue obtained after thyroidectomy. DNA extraction from formalin-fixed paraffin-embedded (FFPE) tissue was conducted using the QIAamp DNA FFPE Tissue kit (Qiagen, Hilden, Germany) according to the manufacturer’s protocol.

### Amplification of BRAFV600E gene

Fifty nanograms of DNA was amplified in a 20-μl reaction solution containing 10 μl of 2× concentrated HotStarTaq Master Mix (Qiagen), including PCR buffer with 3 mM MgCl_2_, 400 μM of each dNTP, and 0.3 μM of each primer pair (exon 15, forward: 5′- ATGCTTGCTCTGATAGGAAAATGA; reverse: 5′- AGCAGCATCTCAGGGCCA). The amplifications were performed under the following conditions: a 15-minute initial denaturation at 95°C; 35 cycles of 30 s at 94°C, 30 s at 58°C, and 45 s at 72°C; and a 10-minute final extension at 72°C. The PCR products were then 2% gel-purified using a QIAgen gel extraction kit (Qiagen).

### Direct sequencing

The DNA templates were processed for the DNA sequencing reaction using the ABI-PRISM BigDye Terminator version 3.1 (Applied Biosystems, Foster, CA, USA) with both forward and reverse sequence-specific primers. Twenty nanograms of purified PCR products were used in a 10-μl sequencing reaction solution containing 1 μl of BigDye Terminator v3.1 and 0.1 μM of the same PCR primer. Sequencing reactions were performed using 25 cycles of 10 s at 96°C, 5 s at 50°C, and 4 minutes at 60°C. The sequence data were generated with the ABI PRISM 3730 DNA Analyzer (Applied Biosystems). The sequences were analysed and compared using Sequencing Analysis 5.1.1. software (Applied Biosystems).

### Statistical analysis

Data analyses were performed with SPSS version 18.0 (SPSS Inc., Chicago, IL, USA). The chi-square test and Fisher’s exact test were used for categorical variables, and Student’s *t*-test for independent samples was used for continuous variables. Continuous variables were presented as the mean ± standard deviation. A multivariate analysis was performed using logistic regression analysis. Differences were considered statistically significant when *P* was <0.05.

## Results

The clinicopathological characteristics of the 327 patients, 268 female (82%) and 59 male (18%), with a mean age of 47.79 ± 11.03 years at the time of surgery, are summarised in Table 1.

**Table 1:** Clinicopathological characteristics of the study population (n = 327)

**Characteristics**	**Value**
Age, years,	
mean ± SD	47.79 ± 11.03
range	
≥45	210 (64.2%)
<45	117 (35.8%)
Sex,	
male	59 (18%)
female	268 (82%)
Tumour size, cm,	
mean ± SD	1.04 ± 0.73
≤1 cm	213 (65.1%)
>1 cm	114 (34.9%)
Extrathyroidal extension	192 (58.7%)
BRAF^V600E^ mutation	241 (73.7%)
Lymph node metastasis	147 (45%)
Central node metastasis	145 (44.3%)
Lateral node metastasis	28 (8.6%)
TNM stage	
I	137 (41.9%)
II	40 (12.2%)
III	121 (37.0%)
IVA	18 (8.9%)
Extent of surgery	
Total thyroidectomy	270 (82.6%)
Hemithyroidectomy	55 (16.8%)
Completion thyroidectomy	2 (0.6%)
Hashimoto thyroiditis	56 (17.1%)

Results are presented as mean ± SD, or number of patients (%). TNM, classification by tumour, nodes and metastases.

The mean tumour size was 1.04 ± 0.73 cm. Extrathyroidal extensions and lymph node metastases were present in 192 patients (58.7%) and 147 patients (45%), respectively.

Total thyroidectomy was performed in 270 patients (82.6%). Other thyroidectomies, including hemithyroidectomies and completion thyroidectomies, were performed in 57 (17.4%) patients. Of the 327 patients with PTC, 56 (17.1%) demonstrated pathological features of Hashimoto’s thyroiditis. In total, 137 patients (41.9%) had stage I disease, 40 (12.2%) had stage II disease, 121 (37.0%) had stage III disease, and 18 (8.9%) had stage IVA disease. The BRAF^V600E^ mutation was detected in the tumours of 241 patients (73.7%).

Table 2 shows the association between BRAF^V600E^ mutation status and the clinicopathological characteristics of the patients. Lymph node metastasis, TNM stage, and multifocality were not significantly associated with the BRAF^V600E^ mutation. However, larger tumour size, extrathyroidal extensions, and histologic type (classic type) concurrent with Hashimoto’s thyroiditis were associated with the BRAF^V600E^ mutation.

**Table 2:** **Univariate analysis of the association between clinicopathological characteristics and the BRAF**^**V600E **^**mutation**

**Variables**	**BRAF**^**V600E **^**mutation**		***P*-value**
	**Positive (n = 241)**	**Negative (n = 86)**	
Age, years,			
mean ± SD	47.63 ± 11.26	48.23 ± 10.41	0.665
≥45	85 (35.3%)	32 (37.2%)	0.747
<45	156 (64.7%)	54 (62.8%)	
Sex,			
male	39 (16.2%)	20 (23.3%)	0.143
female	202 (83.8%)	66 (76.7%)	
Tumour size, cm,			
mean ± SD,	1.09 ± 0.72	0.89 ± 0.75	0.040
≤1 cm	147 (61%)	66 (76.7%)	0.009
>1 cm	94 (39%)	20 (23.3%)	
Extrathyroidal extension,			
yes	152 (63.1%)	40 (46.5%)	0.007
no	89 (36.9%)	46 (53.5%)	
Lymph node metastasis,			
yes	110 (47.6%)	37 (43.5%)	0.518
no	121 (52.4%)	48 (56.5%)	
Central node metastasis,			
yes	110 (47.6%)	35 (41.2%)	0.308
no	121 (52.4%)	50 (58.8%)	
Lateral node metastasis,			
yes	19 (8.2%)	9 (10.6%)	0.512
no	212 (91.8%)	76 (89.4%)	
TNM stage,			
I	96 (41.6%)	41 (48.2%)	0.138
II	33 (14.3%)	7 (8.2%)	
III	91 (37.7%)	30 (34.8%)	
IVA	11 (6.4%)	7 (8.8%)	
Multifocality,			
yes	59 (24.5%)	23 (26.7%)	0.678
no	182 (75.5%)	63 (73.3%)	
Histology,			
classic type	233 (96.7%)	78 (90.7%)	0.027
follicular variant	8 (3.3%)	8 (3.3%)	
Concurrent Hashimoto’s thyroiditis,			
yes	35 (14.5%)	21 (24.4%)	0.037
no	206 (85.5%)	65 (75.6%)	

Results are presented as mean ± SD, or number of patients (%). TNM, classification by tumor, nodes and metastases.

The results of the multivariate analysis are shown in Table 3. The BRAF^V600E^ mutation was not significantly associated with tumour size, extrathyroidal extension, or the histologic type concurrent with Hashimoto’s thyroiditis.

**Table 3:** **Multivariate analysis of the association between clinicopathological characteristics and the BRAF**^**V600E **^**mutation**

**Variables**	**Odds ratio**	**95% CI**		***P*-value**
		**Lower**	**Upper**	
Tumour size (>1 cm)	2.050	0.887	4.736	0.093
Extrathyroidal extension (+)	1.508	0.874	2.602	0.140
Histology (classic type)	2.697	0.926	7.855	0.069
Hashimoto’s thyroiditis	0.584	0.312	1.095	0.093

Of the 327 patients with PTC, 247 (75.5%) received postoperative radioiodine-131 treatment. There was no I-131 avidity in the foci of lateral lymph node metastases.

## Discussion

In 2003, Kimura *et al*. initially reported the BRAF^V600E^ mutation in thyroid carcinomas. Since this discovery, additional research has been conducted to understand the tumorigenic role and clinical importance of this mutation [11-13]. The BRAF^V600E^ mutation has been reported in PTC with a frequency ranging from 29% to 83% in various studies [14], and recent studies have reported differences in the incidence of the BRAF^V600E^ mutation according to region; for example, the incidence of the BRAF^V600E^ mutation was shown to be higher in Korea (range 52% to 87%) in comparison to other countries (range 36% to 65%) [7,8]. The reason for this variation in frequency remains unclear but may be due to geographic factors. For example, some studies have reported higher rates of the BRAF^V600E^ mutation in high iodine intake areas, which may indicate the significance of iodine as a risk factor for the BRAF^V600E^ mutation in PTC [8,15]. Iodine intake is very high in Korea as compared to Western countries, which may explain the high prevalence of the BRAF^V600E^ mutation among Koreans with PTC. The prevalence of the BRAF^V600E^ mutation in PTC patients in our study was 73.7% (241/327), and further studies will be needed to clarify differences in the prevalence of the BRAF^V600E^ mutation between geographic regions.

Many studies have reported the relationship between the BRAF^V600E^ mutation and the clinicopathological characteristics of PTC. However, recent clinical studies have reported controversial results regarding whether the BRAF^V600E^ mutation serves as a prognostic or predictive marker of PTC. Xing *et al*. [16] first reported that the BRAF^V600E^ mutation was associated with a poorer clinicopathological outcome and independently predicted recurrence. These authors therefore concluded that the BRAF^V600E^ mutation may be a useful marker for risk stratification in PTC. Many studies have found that extrathyroidal extensions, lymph node metastases, and advanced stage are the three most common risk factors consistently associated with the BRAF^V600E^ mutation [17-19]. In addition, several studies have reported significant associations between the BRAF^V600E^ mutation and clinicopathological characteristics, such as older age, male sex, tumour size, and an aggressive subtype [17-21].

Although all of the above-mentioned studies were conducted in Western countries, some results from studies conducted in Eastern countries have not been consistent, especially those performed in the Korean population. Ito *et al*. [22] investigated the BRAF^V600E^ mutation in 631 patients with PTC and found that the BRAF^V600E^ mutation was not significantly associated with high-risk biological features, such as lymph node metastasis, extrathyroidal extension, advanced age, distant metastasis at surgery, and advanced stage. Furthermore, the disease-free survival of patients with the BRAF^V600E^ mutation did not differ from that of patients without the BRAF^V600E^ mutation. In addition, other study groups have reported that age distribution, tumour size, extrathyroid extension, multifocality, and staging do not differ significantly between patients with and without the BRAF^V600E^ mutation [7]. Our study found that large tumour size (odds ratio (OR) 2.131, 95% CI 1.266, 3.588) and extrathyroidal extensions (OR 1.964, 95% CI 1.194, 3.232) were associated with the BRAF^V600E^ mutation in a univariate analysis, although no clinicopathological features were associated with the BRAF^V600E^ mutation in a multivariate analysis. Therefore, our results indicate that there may be differences between PTC subjects in Eastern and Western countries.

The prevalence of the BRAF^V600E^ mutation in PTC is distinctly different between subtypes. For example, this mutation is specific to the classic and tall-cell variants of PTC and is rarely found in other types of differentiated thyroid cancer, including follicular variant PTC [13]. We also observed a significant prevalence of the BRAF^V600E^ mutation among patients with classic type PTC311 (95.1%), whereas only 16 patients (4.9%) with the follicular variant PTC harboured the BRAF^V600E^ mutation.

In this study, we found that the BRAF^V600E^ mutation was associated with a lower frequency of concurrent Hashimoto’s thyroiditis in a univariate analysis (*P* <0.05). The BRAF^V600E^ mutation was present in 21 (24.4%) of 86 patients with PTC without Hashimoto’s thyroiditis and in 35 (14.5%) of 241 patients with both PTC and Hashimoto’s thyroiditis. In a previous Korean study [23], the BRAF^V600E^ mutation was detected at a higher frequency among patients with PTC but without Hashimoto’s thyroiditis (90%) as compared to those with PTC and Hashimoto’s thyroiditis (64%). Together, these results suggest that the BRAF^V600E^ mutation is associated with the tumourigenesis of most classic type PTCs and that the somatic BRAF^V600E^ mutation likely acts on a component of Hashimoto’s thyroiditis following PTC as cells age. However, the current study had some inherent limitation. First, the rate of mirocarcinoma was higher (65.1%) in this study, and this condition is known to be associated with low aggressiveness and a more favourable prognosis in most cases. Second, we were not able to examine disease recurrence and describe the long-term follow up results, and the time period of the present study was too short to identify the prognostic value of the BRAF^V600E^ mutation. Thus, a long-term follow up study is needed to identify the association between the prognosis of PTC and the BRAF^V600E^ mutation.

## Conclusions

The prevalence of the BRAF^V600E^ mutation in this study was in accordance with that observed in previous studies. This prevalence was related to several clinicopathological factors, including extrathyroidal extension, large tumour size, classic histologic type, and a low frequency of concurrent Hashimoto’s thyroiditis. However, we failed to establish a correlation with regard to lymph node metastasis, advanced stage, sex, and age. Moreover, there were no independent factors associated with BRAF^V600E^ mutation status. However, it will be necessary to perform a long-term follow up study to identify the clinical usefulness of the BRAF^V600E^ mutation in PTC.

## Abbreviations

FFPE: Formalin-fixed paraffin-embedded; FNAC: Fine needle aspiration cytology; PTC: Papillary thyroid carcinoma; OR: Odds ratio; PCR: Polymerase chain reaction; TNM: Tumour nodes, metastases.

## Competing interests

The authors declare that they have no competing interests.

## Authors’ contributions

WCP participated in the conception and design of the study and carried out the surgery. YSK participated in the data analysis and interpretation and drafted the manuscript. JSK and JSB participated in the collection of the data. All authors read and approved the final manuscript.
